# Cervical Necrotizing Fasciitis: An Institutional Experience

**DOI:** 10.7759/cureus.32382

**Published:** 2022-12-10

**Authors:** Vikas Gupta, Shaila Sidam, Ganakalyan Behera, Aman Kumar, Utkal P Mishra

**Affiliations:** 1 Otorhinolaryngology, All India Institute of Medical Sciences, Bhopal, Bhopal, IND; 2 Otolaryngology - Head and Neck Surgery, All India Institute of Medical Sciences, Bhopal, Bhopal, IND; 3 Otolaryngology, All India Institute of Medical Sciences, Bhopal, Bhopal, IND; 4 Radiology, All India Institute of Medical Sciences, Bhopal, Bhopal, IND

**Keywords:** necrosis, mortality, broad spectrum antibiotics, necrotizing fascitis, fatal, urgent debridement, cervical necrotizing fasciitis

## Abstract

Introduction

Cervical necrotizing fasciitis is an acute, progressive, and rapidly spreading soft tissue infection affecting the fascial planes of the head and neck region. It has high morbidity and mortality rate. In this study, we have reviewed cervical necrotizing fasciitis cases treated in our department and analyzed the various risk factors, laboratory indices, and treatment modalities that affect the prognosis of this deadly disease.

Design and method

This is a retrospective review. We have reviewed the medical records and charts of seven patients hospitalized in our institute with the diagnosis of cervical necrotizing fasciitis between 2015 and 2019.

Results

Of the seven patients, six were male and one was female. The mean age was 49.8 years (range: 38-70 years). Etiology was found to be odontogenic infection in five (71%) cases. The presenting feature in all cases was tender cervical swelling. Intraoperatively, the submandibular triangle was found to be involved in all cases (100%) followed by the carotid triangle in five (71%) cases and the submental triangle in three (42%) cases. The most common comorbidities associated with cervical necrotizing fasciitis were found to be uncontrolled diabetes mellitus and anemia. All patients underwent emergency aggressive surgical debridement and culture-directed broad-spectrum antibiotics (100%). Additional procedures in the form of tracheostomy were required in two (28%) cases and skin grafting in two (28%) cases. One patient in our series developed sepsis with descending mediastinitis. The average hospital stay was 27 days. All the patients survived with no mortality.

Conclusion

Cervical necrotizing fasciitis should be diagnosed early. Early initiation of broad-spectrum antibiotics and aggressive surgical debridement are the two key management strategies that can improve survival. Strict glycemic control and correction of anemia result in a favorable outcome.

## Introduction

Cervical necrotizing fasciitis (NF) is a rapidly progressive infection of cervical fascial planes. It is an acute, difficult-to-diagnose infection, which causes necrosis of the subcutaneous tissue and the superficial fascial planes, leading to widespread gangrene and deep neck space abscess. The rate of tissue destruction can reach up to 2-3 cm/hour [1-2].

NF usually affects the abdomen, groin, perineum, and extremities. Head and neck involvement is seen in 1% to 10% of cases [3]. The most frequent sources of origin of infection are odontogenic or pharyngeal. It is mostly caused by mixed flora of aerobes and anaerobes [3]. This rapidly spreading necrosis leads to early systemic toxicity, which results in potentially fatal complications such as airway compromise, sepsis with multi-organ failure, and descending mediastinitis.

The mortality rate in cervical NF may range from 4% to 50% depending on the virulence of causative organisms and comorbidities [4-5]. Therefore, early diagnosis and emergency management in the form of securing the airway, broad-spectrum intravenous antibiotics, and aggressive surgical debridement are essential to improve overall survival.

We reviewed the medical records of the patients hospitalized in our institute with the diagnosis of cervical NF and tried to summarize the factors affecting overall survival in this fatal disease.

## Materials and methods

We performed a retrospective study on patients admitted to the Department of Otorhinolaryngology-Head & Neck Surgery between January 2015 and December 2019. Institutional Human Ethics Committee approval was obtained with a waiver of consent. Clinical records were searched using the keywords: “cervical necrotizing fasciitis,” “deep neck space infection,” and “neck abscess.” Only clinical or biopsy-proven cases of cervical NF were included in this study. Clinical criteria for inclusion were patients who had tender neck swelling with blackish skin discoloration or intraoperative evidence of cervical fascial necrosis during neck exploration. All other cases of neck abscesses without histopathological evidence of tissue necrosis were excluded from the study. In all patients, an urgent computed tomography scan of the neck and thorax was performed before surgery. Clinical and demographic details including addiction history, comorbidities, laboratory parameters, radiological findings, the extent of surgery, culture sensitivity data, histopathological report, type of antibiotics administered, total duration of hospital stay, postoperative recovery, and outcome of the patients were noted in a clinical proforma. Only seven cases fulfilled the inclusion criteria, among which six were male and one was female. Surgical intervention in the form of neck exploration and aggressive debridement was performed in all the cases by experienced head and neck surgeons. After debridement, the wound was irrigated with injection metronidazole (500mg/100mL) and 10% povidone-iodine solution. Regular wound dressing was done twice daily for 5 days or till no active discharge from the wound was noted. Pus and necrotic tissue were sent for culture sensitivity and histopathological examination. All the patients were started on empirical broad-spectrum antibiotics consisting of injectable amoxicillin clavulanate, metronidazole, and amikacin, which were later on shifted to culture-directed antibiotics. Ancillary procedures performed in the form of tracheostomy and split skin grafting were also noted. All these details were tabulated, and factors affecting survival in cervical NF were analyzed.

## Results

Among seven cases of cervical NF, six patients were male and one was female. Addiction history revealed addiction to tobacco chewing in four (57.14%) patients and smoking along with alcohol intake in three patients (42.8%). The mean age was 49.8 years (range: 38-70 years), and six of our patients were above 40 years. The commonest comorbidity associated with cervical NF was anemia in seven cases and type II diabetes mellitus (DM) in five cases. The source of infection was found to be odontogenic in five cases and in two cases it was due to peritonsillar abscess (Table 1).

**Table 1:** Details of factors affecting survival in cervical necrotizing fasciitis E. coli, Escherichia coli; MRSA, methicillin-resistant Staphylococcal aureus; NLR, neutrophil-to-lymphocyte ratio; PLR, platelet-to-lymphocyte ratio

	Case 1	Case 2	Case 3	Case 4	Case 5	Case 6	Case 7
Age/sex	51/male	58/male	45/male	42/male	45/male	70/female	38/male
Etiology	Peritonsillar abscess	Dental origin	Dental origin	Dental origin	Peritonsillar abscess	Dental origin	Dental origin
Leukocytosis (%)	Yes	Yes	No	Yes	Yes	Yes	Yes
Neutrophilia (%)	Yes	Yes	No	Yes	Yes	Yes	Yes
NLR	Mild	Mild	Mild	Mild	Moderate	Moderate	Moderate
PLR	42	26	23	54	33	41	28
Anemia (Hb gm%)	Yes	Yes	Yes	Yes	Yes	Yes	Yes
Hyperglycemia	Yes	No	Yes	Yes	Yes	No	Yes
Bacteriology	*E. coli*, MRSA, groupA* Streptococcus*	Methicillin-sensitive* Staphylococcus aureus*	*E. coli*, MRSA, group A *Streptococcus*	*E. coli*, MRSA, group A* Streptococcus*	*E. coli*, MRSA, groupA* Streptococcus*	*E. coli*, MRSA, groupA* Streptococcus*	Aerobic gram +ve cocci + gram -ve bacilli
Culture sensitivity	Linezolid	Ceftriaxone, amikacin, metronidazole	Linezolid	Linezolid	Linezolid	Linezolid	Piperacillin, vancomycin, amikacin
Added procedures	Tracheostomy	Split skin grafting	Split skin grafting	Split skin grafting	Split skin grafting	Split skin grafting	Tracheostomy
Complication	No	No	No	No	No	No	Sepsis, Descending Mediastinitis
Length of stay	25	15	41	15	23	30	45
Outcome	Survived	Survived	Survived	Survived	Survived	Survived	Survived

**Figure 1:**
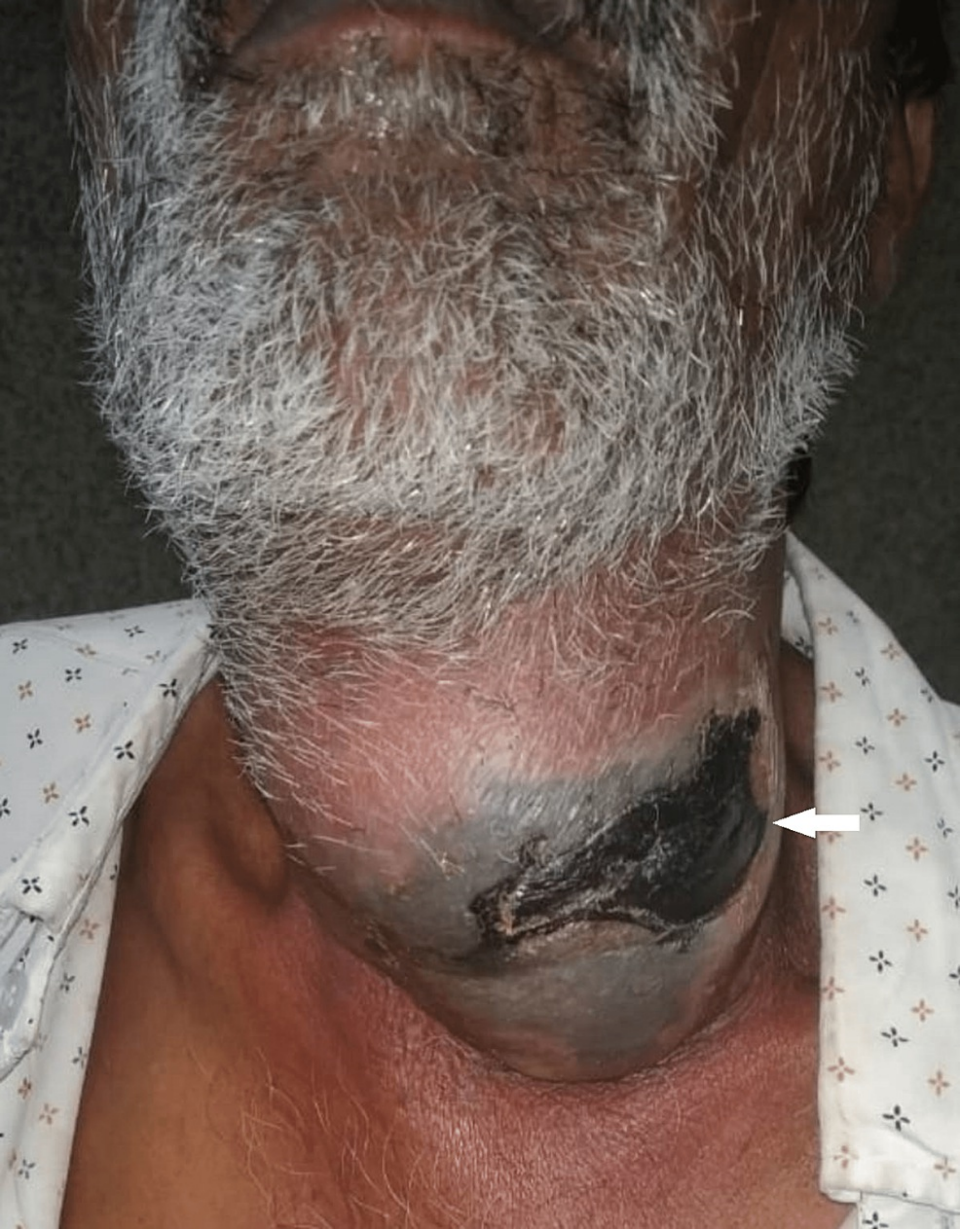
Swelling of the neck with blackish discoloration of overlying skin.

**Figure 2:**
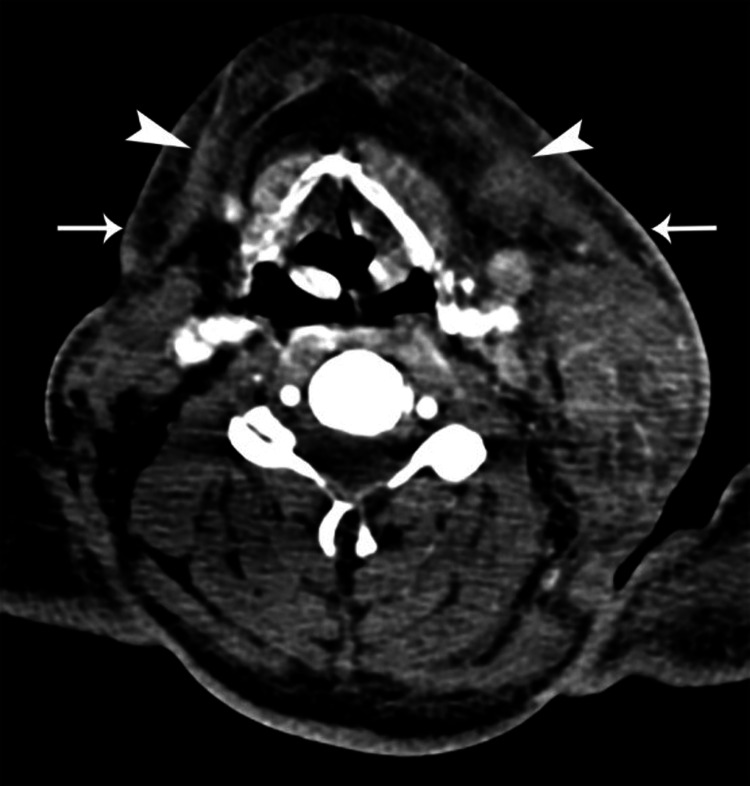
CT scan of the neck showing diffuse thickening of cutis, subcutis (arrows), and platysma (arrowheads) with fat stranding.

**Figure 3:**
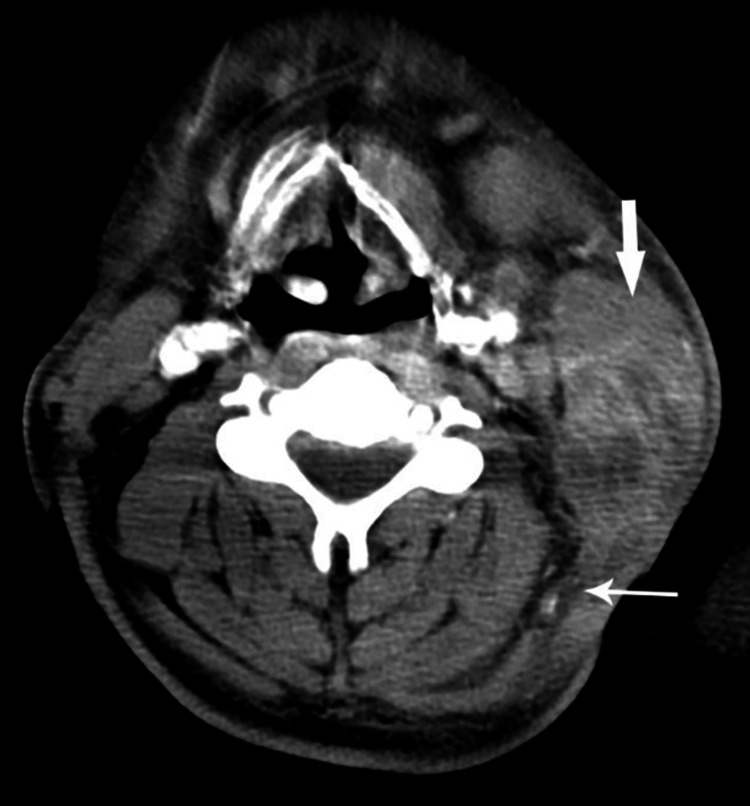
CT scan of the neck shows thickening and enhancement of deep cervical fascia (fasciitis) (thin arrow) enlargement and heterogenous enhancement of muscles (myositis) (thick arrow).

**Figure 4:**
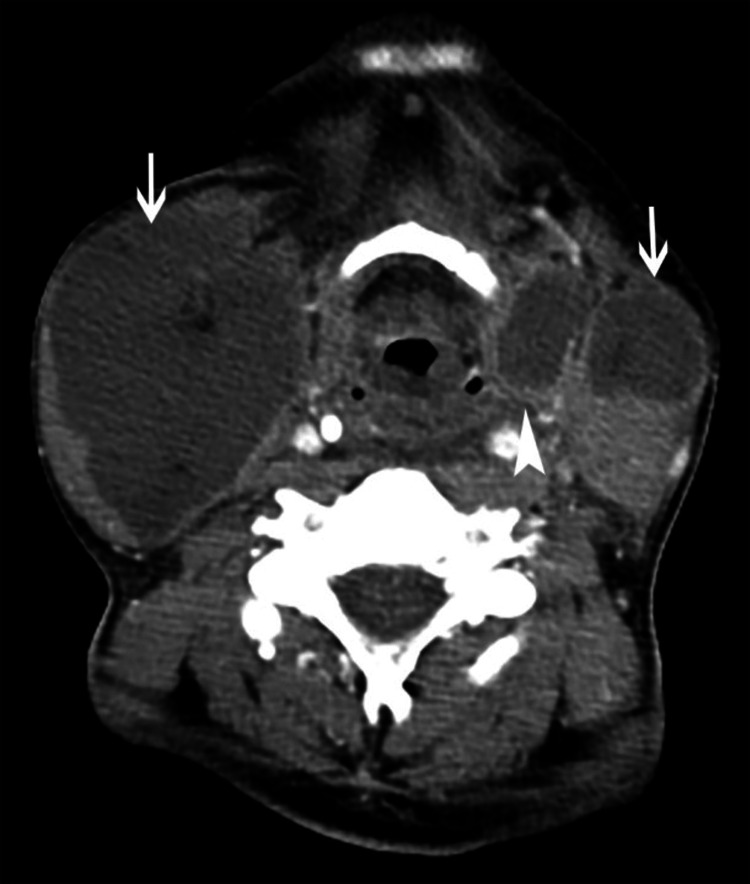
CT scan of the neck showing extensive myonecrosis involving bilateral sternocleidomastoid muscles (arrows). There is a paralaryngeal abscess (arrowhead) extending beyond thyrohyoid membrane in larynx.

Presenting symptoms in all seven patients were tender neck swelling. Four of the patients had developed blackish skin discoloration (Figure 1), and three patients had associated dysphagia. Emergency contrast-enhanced computed tomography (CECT) scan of the neck and thorax was done in all patients. Five patients showed air streaks in cervical soft tissues, which are highly suggestive of NF. CECT also revealed diffuse thickening of the skin, subcutaneous tissues, and platysma with fat stranding, consistent with cellulitis (Figure 2), thickening and enhancement of deep cervical fascia consistent with fasciitis (Figure 3), diffuse enlargement, and irregular enhancement of neck muscles consistent with myositis (Figure 4). Two patients had non-enhancing collections within sternocleidomastoid muscles, suggestive of myonecrosis and abscess formation (Figure 4). Radiological assessment of cervical space involvement revealed involvement of the submandibular triangle in all the cases, followed by the carotid triangle, submental triangle, and parapharyngeal space. Descending mediastinitis was found in one patient with evidence of numerous gas spaces occupying superior mediastinum in CECT of the thorax.

Analysis of laboratory indices revealed leukocytosis and neutrophilia in six cases. Anemia was found in all cases, and hyperglycemia, hypoalbuminemia, and hyponatremia were observed in five cases. The neutrophil-lymphocyte ratio showed a scattered distribution which was mild in four and moderate in three cases.

All cases underwent urgent neck exploration with aggressive debridement of necrotic tissues under general anesthesia. In two cases, the airway was secured with pre-procedure tracheostomy. The mean interval from initial presentation to neck exploration was 24 hours. The wound healed well by secondary intention in two patients and in five patients split skin grafting was required to cover the skin defect, at a later date.

The pus and blood culture results revealed multibacterial growth of *E. coli*, methicillin-resistant *staphylococcus aureus* (MRSA), and group A *streptococcus* in five patients. MRSA was isolated in the pus of one patient. Mixed aerobic gram-positive cocci and gram-negative bacilli were detected in the pus from the patient with descending mediastinitis. MRSA isolates in five patients were found to be sensitive to linezolid. Therefore, they were switched to linezolid after three days of empirical antibiotic therapy.

Histopathological examination in all cases revealed inflammatory granulocytes, vasculitis, necrosis, and collagen fragmentation.

One patient developed sepsis during the treatment, which was managed in the intensive care unit. All the patients have survived in our study with a total hospital stay ranging from 15 to 45 days with an average stay of 27 days. The duration of stay seems to be longer in the elderly, with uncontrolled DM, and in case of requirement of split skin grafting for wound closure.

## Discussion

The term “necrotizing fasciitis” was coined by Wilson in 1952 for necrotizing soft tissue infections [1]. NF is a rapidly progressive fulminant soft tissue infection, which can be fatal if not treated timely and adequately. It begins in superficial fascial planes and extends into the deep fascial planes causing widespread necrosis due to microvascular occlusion. It has a rare occurrence in the cervical region because of its robust blood supply [3].

The most common cause of cervical NF is odontogenic and pharyngeal infections. Mostly, the infections originate from the second and third molar tooth, as their roots extend into the alveolus below the insertion of the mylohyoid muscle [5]. This provides a direct route for the spread of dental infection into the submandibular space. In the neck, submandibular and carotid triangles are commonly involved as they are the major areas of lymphatic drainage [6]. Around 60% of our cases had a dental infection with a predominant involvement of submandibular (100%) and carotid (70%) triangles.

Uncontrolled DM remains a predisposing factor in around 40% to 60% of cases in the published data [1-2]. In our series also, around 72% of patients were having poorly controlled DM. Other factors are alcohol intake, malnutrition, and chronic liver failure [7]. NF is polymicrobial, where synergism between the microbes helps to consume oxygen from tissues, which makes it favorable for anaerobes. The anaerobes provide metabolic substrates to enhance the virulence of aerobes [8].

NF begins with progressive liquefaction of subcutaneous fat and connective tissue which is mediated by the collagenase and hyaluronidase produced by group A streptococci. This results in the separation of skin from the underlying soft tissue producing brownish edematous fluid, which is pathognomonic “dishwater pus” [8]. It is followed by the disintegration of fascial planes, venous thrombosis, and inflammatory cell infiltration. This results in the spread of infection to deep cervical spaces with vascular compromise because of endarteritis obliterans of nutrient vessels. Widespread vascular compromise leads to skin necrosis and gangrene formation [9].

Wang et al. have described a clinical triad in cervical NF. NF presents with a triad of local pain, swelling, and erythema (stage 1), followed by blistering and bullae (stage 2), and crepitus, skin anesthesia, and necrosis (stage 3) [3]. The most common presenting feature in this study is neck swelling and necrosis of the skin. There is an increase in glucose levels due to increased gluconeogenesis from protein, resulting in hypoproteinemia [1]. It correlates with our series showing hypoalbuminemia in five patients. Hyperglycemia impairs the leukocyte function and suppresses the host immune system. The result is fewer circulating lymphocytes and T cells. Hence, antibody response is compromised along with polymorphonuclear cell function, which makes them less responsive to infection [7]. It may affect the course of soft tissue infection, increasing the spaces of neck involvement, risk of complication, and morbidity increases. Hence, blood sugar monitoring and strict glycemic control are utmost, which can affect the prognosis.

Bacterial infection, inflammation, thrombosis, and necrosis tend to raise the inflammatory markers [1]. The reliable indicators are C-reactive protein (CRP), creatinine, hemoglobin, leucocyte count, sodium, and serum glucose [10]. As the inflammation becomes severe, CRP and white cell count tend to increase, while hemoglobin and albumin tend to decrease [11]. According to our study, the factors that can affect the course of illness are advanced age, anemia, uncontrolled hyperglycemia, presence of complications, and multiple neck space involvement. The neutrophil-lymphocyte ratio is a good stress indicator as well as an inflammatory marker, which can be used as an indicator of systemic inflammation. In our cases, neutrophil-lymphocyte ratio significantly decreased post-surgery from a high initial value to a low post-surgery value. It helped us in predicting the outcome of the disease [12].

Cross-sectional imaging plays a vital role in making a decision and in assessing the extent, severity, and source of infection in some cases. Plain radiographs have a limited role in serving that purpose and are usually performed to look for air in cervical soft tissues [13]. CECT is an excellent modality to assess the depth and extent of the infection [14]. On CECT, thickening of the skin and immediate subcutaneous tissues with fat stranding is suggestive of cellulitis. Areas of irregular enhancement and fluid collections may be seen. However, the involvement of deeper tissue is suggestive of NF. It may be seen as the thickening and enhancement of deep cervical fascia. Muscular involvement may be seen on CT, with enlargement and hyperenhancement of muscles. Myonecrosis is seen as a non-enhancing area of low attenuation. Abscess formation in NF is often transspatial. In addition, the mediastinal extension of infection can be seen in the form of mediastinal fat stranding or fluid collections.

Treatment involves securing the airway and aggressive surgical debridement, which can halt the spread and release of inflammatory mediators responsible for the systemic complication [3]. All our patients underwent urgent surgical debridement with two patients additionally requiring tracheostomy. Repeated debridement may be needed along with a combination of antibiotic therapy and intensive care support. Reconstruction should be undertaken after the infection has been eliminated and healthy granulating tissue is seen in the wound bed [15].

Delayed recognition, underestimation of disease extent, and reluctance to aggressively debride soft tissue to avoid disfigurement might lead to undertreatment [16]. This will promote the spread of infection and worsen the outcome. Hence, the aim is regular and frequent debridement to improve the bioavailability of drugs in devitalized tissue and to remove all necrotic tissue [9]. It also helps to drain the loculated collection in the fascial planes till viable tissue is encountered, along with frequent wound dressing.

Infection from the head and neck can spread to the mediastinum via retropharyngeal or prevertebral space or along the carotid sheath, which can cause complications such as fulminant mediastinitis, septicemia, pleural effusion, airway obstruction, rupture of major vessels, or respiratory failure [1]. In our series, only one patient developed septicemia.

Death may occur due to sepsis in early cases and multi-organ failure or respiratory failure in late cases.

## Conclusions

Cervical NF is an uncommon, progressive, life-threatening disease of the soft tissue and the fascial planes. The diagnosis of NF can be made faster with detailed clinical examination, CECT, and laboratory findings. Securing the airway, early and aggressive surgical debridement, and broad-spectrum antibiotic, along with intensive nutritional and hemodynamic support remain the key to the successful management of cervical NF.
